# EGFR inhibitors suppress house dust mite allergen Der pII induced inflammation in monocytes and macrophages

**DOI:** 10.3389/falgy.2026.1748679

**Published:** 2026-02-06

**Authors:** Ya-Hui Chiang, I-Lun Hsin, Ping-Ju Chen, Hui-Yi Chang, Jiunn-Liang Ko, Ko-Huang Lue, Yu-Fan Liu

**Affiliations:** 1 Institute of Medicine, Chung Shan Medical University, Taichung, Taiwan; 2 Division of Chest Medicine, Department of Internal Medicine, Cheng Ching Hospital, Taichung, Taiwan; 3 Department of Nursing, National Taichung University of Science and Technology, Taichung, Taiwan; 4 Institute and Department of Food Science, Central Taiwan University of Science and Technology, Taichung, Taiwan; 5 Department of Biomedical Sciences, Chung Shan Medical University, Taichung, Taiwan; 6 Department of Medical Genetics, National Taiwan University Hospital, Taipei, Taiwan; 7 School of Medicine, Chung Shan Medical University, Taichung, Taiwan; 8 Division of Medical Oncology, Department of Internal Medicine, Chung Shan Medical University Hospital, Taichung, Taiwan; 9 Division of Allergy, Asthma and Rheumatology, Department of Pediatrics, Institute of Allergy, Immunology, and Rheumatology, Chung Shan Medical University Hospital, Taichung, Taiwan

**Keywords:** *Dermatophagoides pteronyssinus*, EGFR-TKIs, IL-6, IL-8, macrophage, monocyte

## Abstract

**Introduction:**

Allergic asthma, often triggered by house dust mites (HDMs), is characterized by airway inflammation, mucus hypersecretion, and airway hyperresponsiveness. Among the major HDM allergens, Der pII plays a significant role in promoting inflammation. This study investigates the role of epidermal growth factor receptor (EGFR) inhibitors in modulating Der pII-induced cytokine production and inflammation in human immune cells.

**Methods:**

Recombinant GST-Der pII protein was expressed and purified for subsequent studies. Human peripheral blood mononuclear cells (HPBMC), THP-1 monocytes, THP-1-derived macrophages, and pulmonary alveolar macrophages (NR8383) were exposed to Der pII, followed by treatment with EGFR inhibitors AZD-9291 and Tarceva. Enzyme-linked immunosorbent assay (ELISA) was used to detect the expressions of IL-6 and IL-8. Nitric oxide (NO) levels were determined using the Griess Reagent System.

**Results:**

Der pII significantly induced pro-inflammatory cytokines, including IL-6, IL-8, and TNF-α in HPBMC and THP-1 cells. Both EGFR inhibitors reduced the secretion of IL-6 and IL-8 in these cell types. In THP-1 macrophages, AZD-9291 suppressed IL-6 expression and CD14/CD36 macrophage markers. Moreover, AZD-9291 significantly inhibited NO production in alveolar macrophages.

**Conclusions:**

These findings suggest that EGFR plays a critical role in mediating Der pII-induced inflammation, and EGFR inhibitors may represent a potential therapeutic approach for controlling HDM-induced allergic inflammation.

## 1 Introduction

Allergic asthma affects over 300 million people globally. Its main features include eosinophilic infiltration in the airway, excessive mucus secretion, and airway hyperresponsiveness. Literature indicates that allergic-specific T helper 2 (Th2) cells and related cytokines regulate allergic airway inflammation and stimulate mucus overproduction, further causing airway hyperresponsiveness (1, 2). Among the major causes of asthma, house dust mites are the most significant, affecting 85% of asthma patients. More than 50% of children and adolescents are sensitive to house dust mites (3, 4). Long-term exposure to mite allergens not only causes asthma but also contributes to atopic dermatitis and allergic rhinitis (5). According to the European Community Respiratory Health Survey (ECRHS), the prevalence of mite-related disease in 15 developed European countries was 21.7% (6).

According to Taiwan's Environmental Protection Administration, common allergy-causing mites thrive in household environments. These mites are tiny (∼0.2 mm), preferring temperatures between 22 and 26°C and humidity levels of 70%–80%, feeding primarily on human skin flakes. Research shows that *Dermatophagoides pteronyssinus* (Der p) is the most predominant dust mites in Taiwan (7). A 2006 study by Prof. Lü Kuan's team examined 462 atopic allergic children (ages 2–16) in central Taiwan. Over 80% were sensitive to various mites, especially Der m (79.5%), Der p (90.2%), and Der f (88.9%) (8). House dust mite allergens trigger antigen-presenting cells, driving Th2 activation, IgE production, and immune cell buildup in the lungs, nasal passages, and sensitive skin (9). As of now, the WHO/IUIS Allergen Nomenclature Subcommittee lists 36 allergen groups for Der p (up to Group 36), among which Groups 1, 2, and 23 are major IgE-binding proteins. Groups 1, 2, 5, 7, and 10 together account for over 80% of allergen reactivity in patient sera (10).

Monocytes and macrophages play a vital role in various inflammatory environments due to their plasticity and ability to infiltrate inflamed tissues. Bone marrow–derived blood monocytes can adhere to the vascular endothelium and migrate into tissues in response to inflammatory stimuli (11–13). Alveolar macrophages, located in the alveolar spaces of the lungs, play a key role in eliminating allergens and maintaining immune balance in the pulmonary environment (14). Chemokines such as CCL2 and MCP-1 play crucial roles in regulating the migration and infiltration of monocytes and natural killer (NK) cells (15, 16). Infected-site macrophages use membrane receptors such as CD36, CD14, and TLR4 to recognize pathogens, phagocytose them, and then eliminate internal debris (17).

Our previous research demonstrated that EGFR inhibitors alleviate Der p2-induced inflammation in human bronchial epithelial cells (18). In this study, HPBMC, THP-1 monocytes and THP-1 macrophage were used to investigate Der pII-induced inflammation. We found that EGFR inhibitors inhibit the Der pII-induced IL-6, IL-8, and NO production in HPBMC, monocytes and macrophages. This is the first study to exam the function of EGFR inhibitors on Der pII-induced inflammation in monocytes and macrophages.

## 2 Materials and methods

### 2.1 Cell culture and chemicals

THP-1 cells (ATCC, TIB-202) and NR8383 cells (ATCC, CRL-2192) were obtained from the American Type Culture Collection. THP-1 cells were cultured in RPMI 1640 Medium (GIBCO, 31800022) supplemented with 10% fetal bovine serum. NR8383 cells were cultured in Ham's F12K (GIBCO, 21127022), and supplemented with 15% fetal bovine serum. AZD-9291 MCE (HY-15772) (22289) was purchased from MedChemExpress (New Jersey). Tarceva was purchased from OSI Pharmaceuticals (Northbrook, Illinois).

### 2.2 Purification of GST-Der PII1-129 proteins

Escherichia coli protein expression system was used to express the recombinant GST-Der PII1-129. The detailed protocol for the purification has been described previously (18).

### 2.3 Enzyme-linked immunosorbent assay (ELISA)

After treatment, the medium was collected for ELISA analysis. Human IL-6 ELISA MAXTM Deluxe Set (BioLegend #430505) and Human IL-8 ELISA MAXTM Deluxe Set (BioLegend #431540) were performed to analyze the IL-6 and IL-8 expression, respectively. The expressions of IL-6 and IL-8 by ELISA were following the protocol of manufacturer's instructions.

### 2.4 Nitric oxide (NO) assay

Nitric oxide (NO) levels in cell culture supernatants were determined using the Griess Reagent System. Following stimulation, 50 μL of each collected supernatant was transferred to individual wells of a 96-well plate. An equal volume (50 μL) of Sulfanilamide Solution was then added to each well and incubated at room temperature for 5–10 min. Subsequently, 50 μL of NED Solution was added, followed by incubation in the dark at room temperature for another 5–10 min. If NO was present, a purple-blue color developed, and the absorbance was measured at 520 nm using an ELISA reader. All experiments were performed in triplicate, and NO concentrations were calculated by comparison with a standard curve.

### 2.5 Statistical analysis

One-way ANOVA and Tukey's *post hoc* test by Predictive Analytics SoftWare (PASW) Statistics 18 were performed to conduct the statistical comparisons between two groups. Values of *p* < 0.05 were considered significant. Data are presented as mean ± SD.

## 3 Results

### 3.1 Expression of GST-Der pII recombinant protein

**Figure 1:**
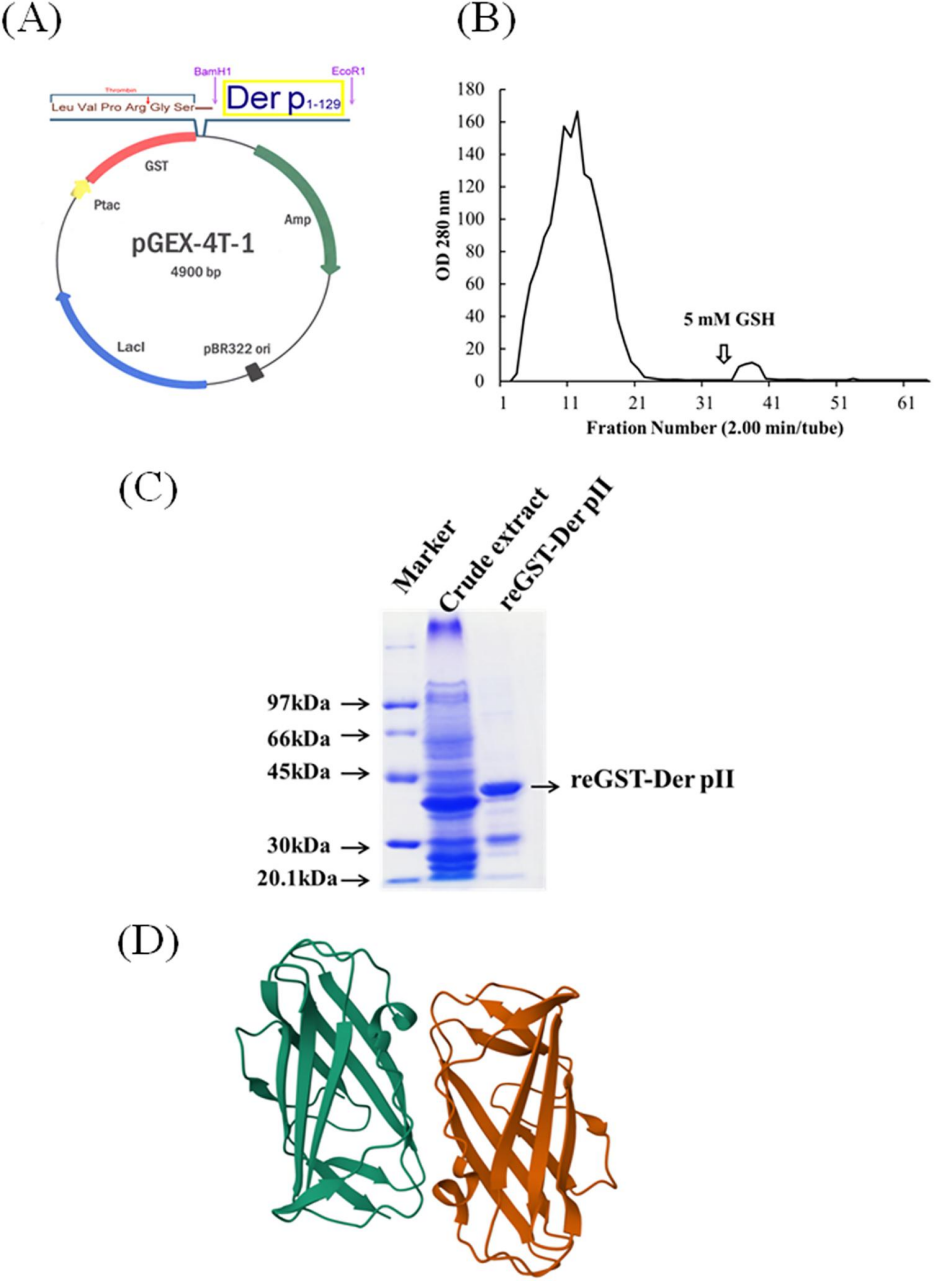
Construction of the expression plasmid pGEX-Der pII and purified the Der pII1-129. **(A)** The gene mapping of pGEX-Der pII containing Der pII coding region 1–129 amino acid. **(B)** The fusion protein was purified by affinity chrmatograph with a Glutathione-Sepharose 4 Fast Flow column. **(C)** SDS-PAGE analyss of fusion protein and purified reDer pII 1–129. **(D)** Protein structure of Der pII (1KTJ) from RCSB Protein Data Bank (RCSB PDB).

To express the Der pII, the gene of Der pII was cloned into pGEX-4t-1 expression vector, in order to create a glutathione-S-transferase (GST) fusion protein with the N-terminus of Der pII (Figure 1A). The GST-Der pII was induced by IPTG and purified (Figures 1B,C). The protein structure of Der pII (1KTJ) from RCSB Protein Data Bank (RCSB PDB) was shown as Figure 1D.

### 3.2 Effect of Der pII on cytokine induction in human peripheral blood mononuclear cells

**Figure 2:**
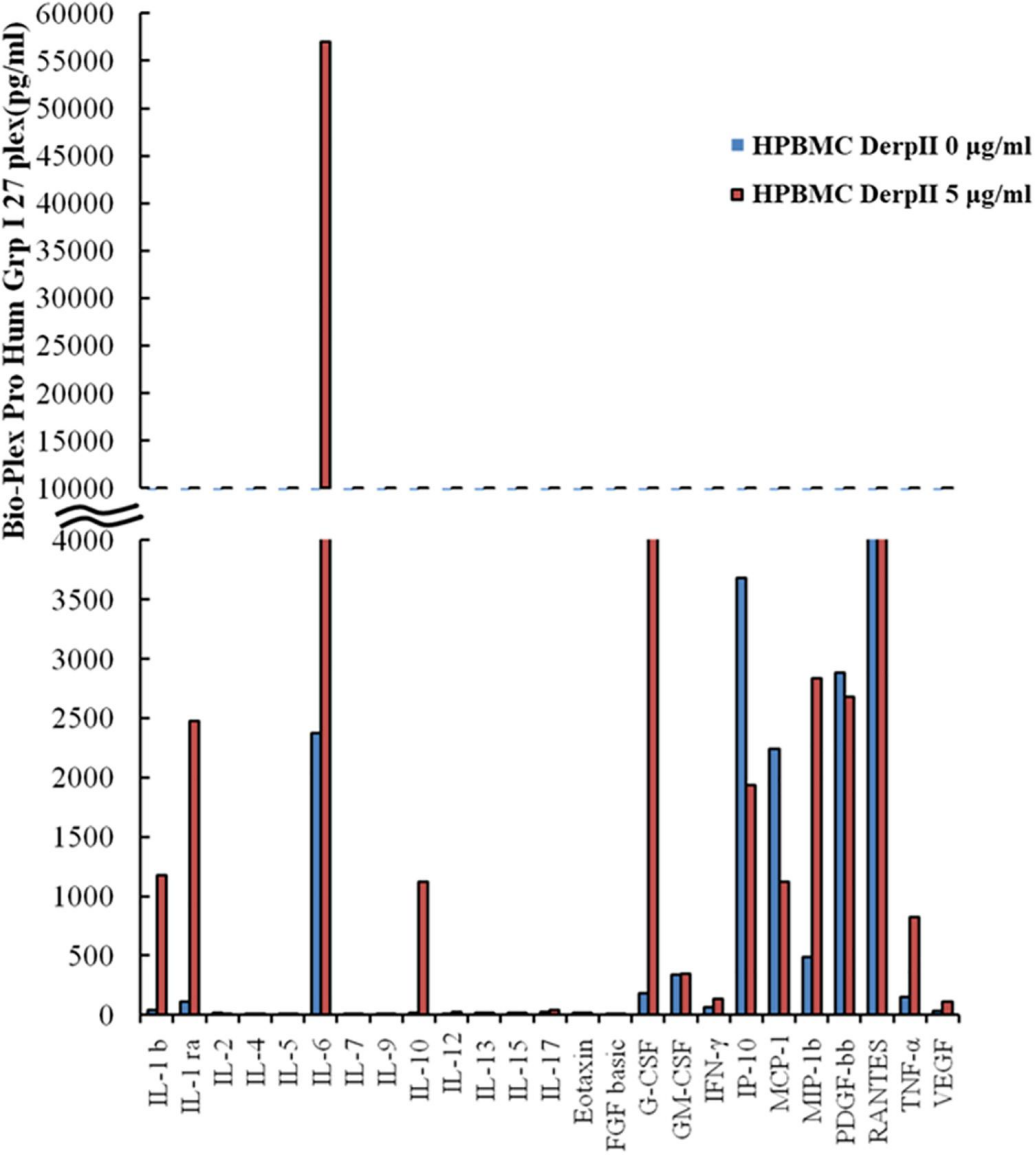
Analysis the production of varies cytokines in Der pII-stimulated HPBMC cells by Bio-Plex Pro human cytokine 27-Plex immunoassay. HPBMC cells (2 × 10^6^cells/well of 24 well) were treated with GST-Der pII (5 μg/mL) for 48 h and the medium was analyzed by Bio-Plex Pro human Cytokine 27-Plex immunoassay.

To examine the effect of Der pII on cytokine production, human peripheral blood mononuclear cells (HPBMCs) were stimulated with Der pII, and cytokine levels in the culture supernatant were assessed using the Bio-Plex Pro Human Cytokine 27-Plex Immunoassay. As shown in Figure 2, Der pII markedly induced the production of interleukin-1 beta (IL-1β), interleukin-1 receptor antagonist (IL-1ra), interleukin-6 (IL-6), interleukin-10 (IL-10), granulocyte-colony stimulating factor (G-CSF), macrophage inflammatory protein-1β (MIP-1β), and tumor necrosis factor-α (TNF-α). To explore potential interactions between EGFR and these Der pII-induced cytokines, the STRING database was utilized. Analysis revealed direct protein–protein associations between EGFR and IL-6, G-CSF, TNF-α, IL-1β, and IL-10 (Supplementary Figure S1).

### 3.3 EGFR inhibitors suppressed Der pII-induced IL-6 and IL-8 expression in HPBMC

**Figure 3:**
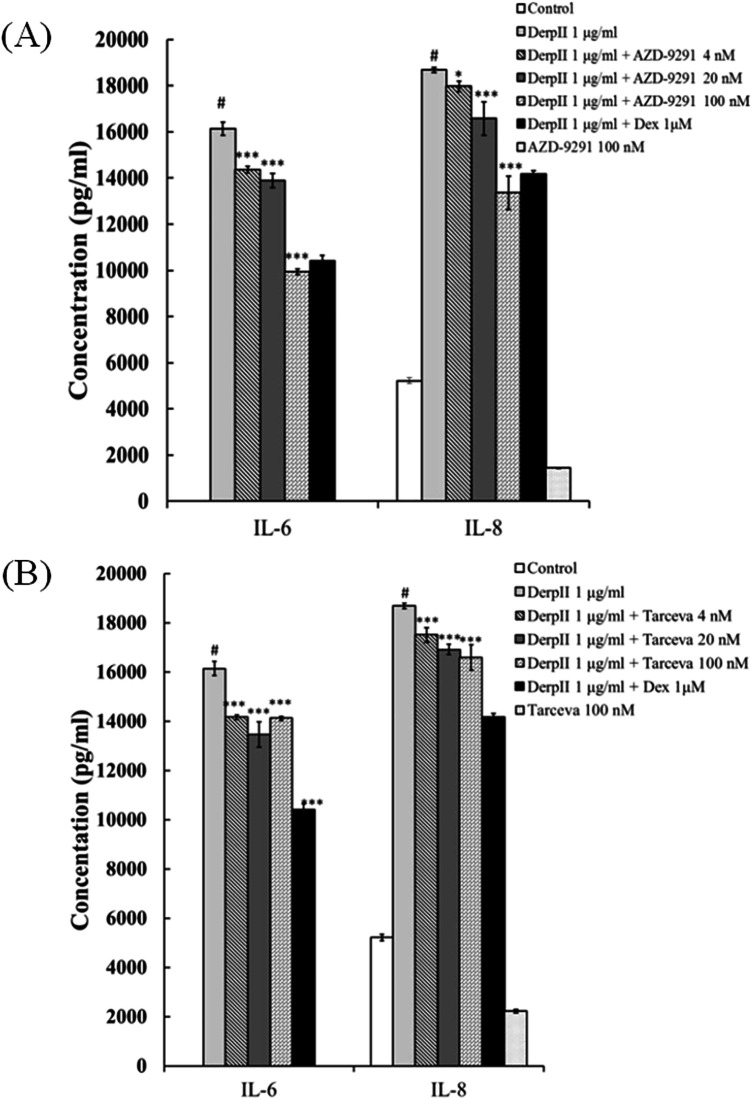
Analysis the production of IL-6 and IL-8 in Der pII-stimulated HPBMC. HPBMC (2 × 10^6^ cells/well of 24 well) were pre-treated with **(A)** AZD-9291 **(B)** Tarceva for 2 h followed by stimulated Der pII (1 μg/mL) for 24 h. Conditioned media were measured amounts of secreted IL-6 and IL-8 by ELISA. The data are expressed as mean ± SD. The symbols (#) indicated significant differences as compared with control, and (*) as compared with Der pII alone. *p < 0.05, **p < 0.01,***p < 0.001.

To evaluate the role of EGFR in IL-6 and IL-8 induction by Der pII, AZD-9291 and Tarceva, two EGFR inhibitors, were used to inhibit the function of EGFR. As shown in Figure 3, Der pII significantly stimulated the secretion of IL-6 and IL-8. Both AZD-9291 and Tarceva significantly reduced the IL-6 and IL-8 induction by Der pII. Treatment with 100 nM AZD9291 reduced Der pII–induced IL-6 and IL-8 production by approximately 37% and 25%, respectively. 100 nM Tarceva treatment reduced Der pII–induced IL-6 and IL-8 production by approximately 13% and 11%, respectively. Dexamethasone (Dex) was used as a positive control in inhibiting IL-6 and IL-8 induction.

### 3.4 Effect AZD-9291 on Der pII-induced mRNA expressions in THP-1 monocytes and THP-1 macrophages

**Figure 4:**
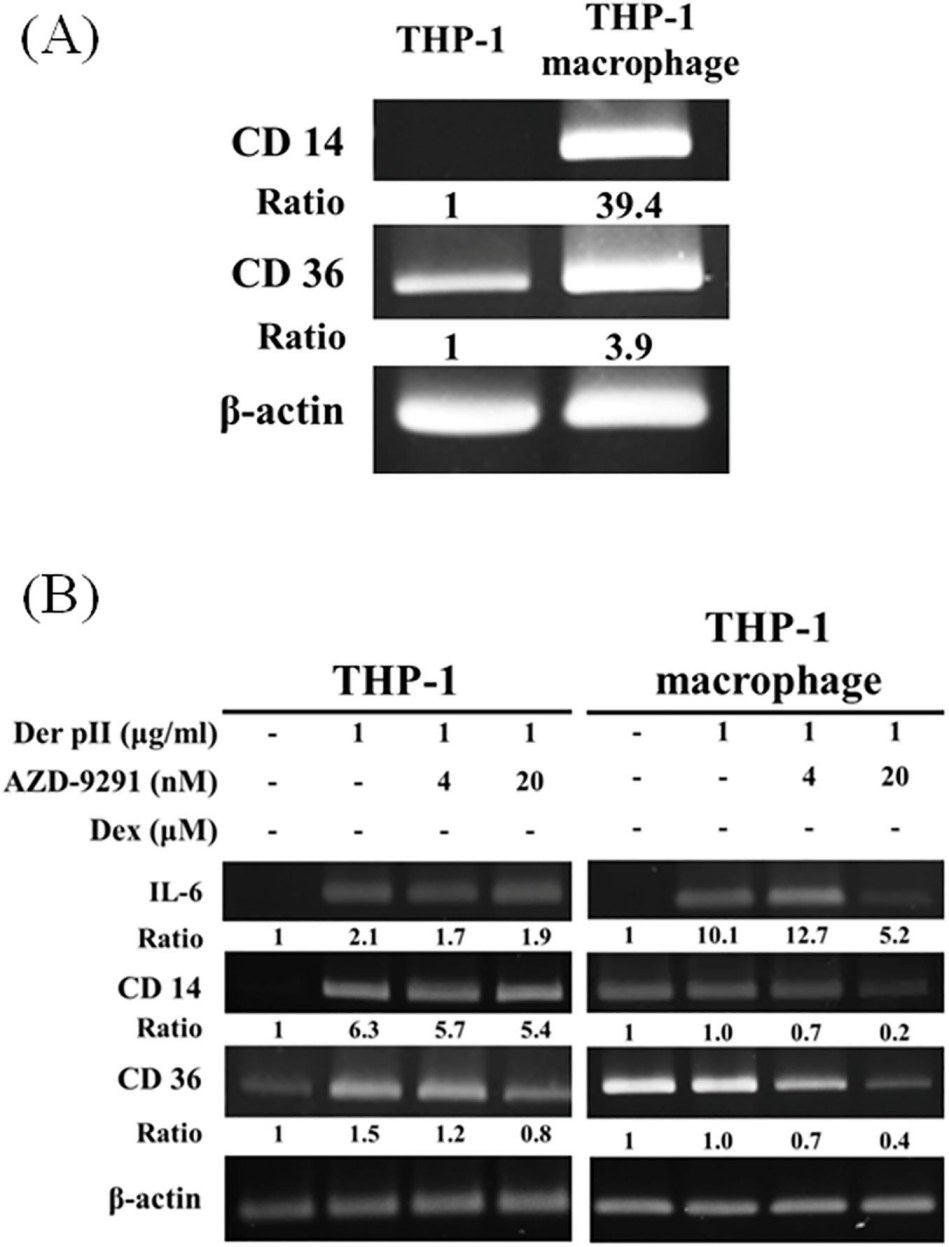
The effects of AZD-9291 on Der pII–stimulated human macrophage. **(A)** Gene expressions of CD14 and CD36 in THP-1 and THP-1 macrophage. **(B)** THP-1 (1 × 10^6^ cells/6 cm dish) and THP-1 macrophage (1 × 10^6^ cells/3.5 cm dish) were pre-treated with AZD-9291 for 2 h followed by stimulated Der pII (1 μg/mL) for 24 h. Gene expressions of IL-6 and IL-8 were analyzed by RT-PCR.

To investigate the effect of Der pII on monocyte and macrophage, THP-1, a monocyte cell line, and THP-1 derived macrophage were used to investigation. After PMA treatment, the THP-1 cells were differentiated into M0 macrophage with increasing of CD14 and CD36, the markers of M0 macrophage (Figure 4A). In THP-1 cells, Der pII increased the gene expressions of IL-6, CD14, and CD36. Der pII induced higher IL-6 expression in THP-1 macrophages compared to THP-1 monocytes (Figure 4B). AZD-9291 reduced the gene expressions of IL-6, CD14, and CD36 in THP-1 macrophages (Figure 4B).

### 3.5 EGFR inhibitors suppressed Der pII-induced IL-6 and IL-8 expression in THP-1 monocyte and THP-1 macrophage

**Figure 5:**
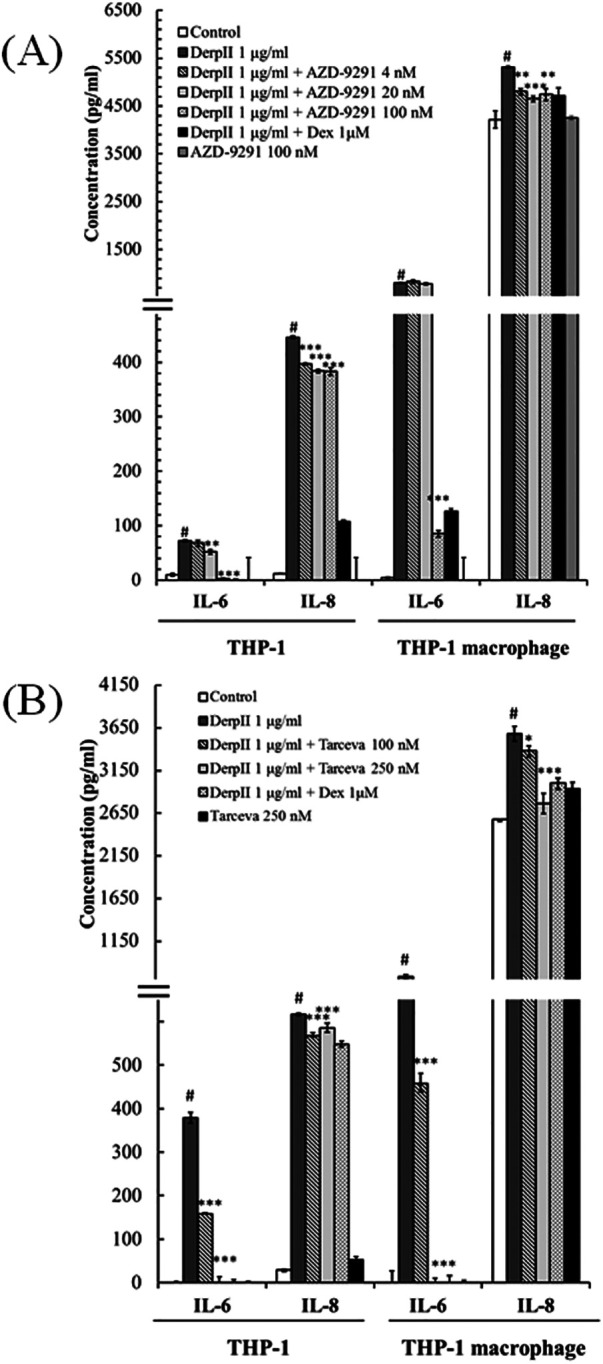
The effects of AZD-9291 on Der pII–stimulated inflammation in human macrophage. THP-1 (1 × 10^6^ cells/6 cm dish) and THP-1 macrophage (1 × 10^6^ cell/3.5 cm dish) were pre-treated with **(A)** AZD-9291 (4, 20 and 100 nM) or **(B)** Tarceva (100 and 250 nM) for 2 h followed by stimulated Der pII (1 μg/mL) for 24 h. Conditioned media were measured amounts of secreted IL-6 and IL-8 by ELISA. The data are expressed as mean ± SD. The symbols (#) indicated significant differences as compared with control, and (*) as compared with Der pII alone. *p < 0.05, **p < 0.01,***p < 0.001.

To examine the involvement of EGFR in Der pII–induced IL-6 and IL-8 expression in THP-1 cells and THP-1 macrophages, EGFR function was inhibited using AZD9291 and Tarceva. In Supplementary Figure S2, we found that 100 nM AZD-9291 or 250 nM Tarceva did not alter the cell viability of THP-1 macrophage with Der pII treatment. Furthermore, AZD-9291 and Tarceva reduced Der pII-induced EGFR activation (Supplementary Figure S3). As shown in Figure 5, Der pII significantly activated the secretion of IL-6 and IL-8 in THP-1 and THP-1 macrophage. Higher expressions of IL-6 and IL-8 were induced by Der pII in THP-1 macrophages compared to THP-1 cells. AZD-9291 and Tarceva significantly reduced the IL-6 and IL-8 induction by Der pII. Treatment with 100 nM AZD9291 decreased Der pII–induced IL-6 and IL-8 production by approximately 93% and 15% in THP-1 macrophages, respectively. 250 nM Tarceva treatment decreased Der pII–induced IL-6 and IL-8 production by approximately 99% and 20%, respectively. Dexamethasone (Dex) was used as a positive control in inhibiting IL-6 and IL-8 induction.

### 3.6 AZD-9291 suppressed Der pII-induced NO production in pulmonary alveolar macrophage

**Figure 6:**
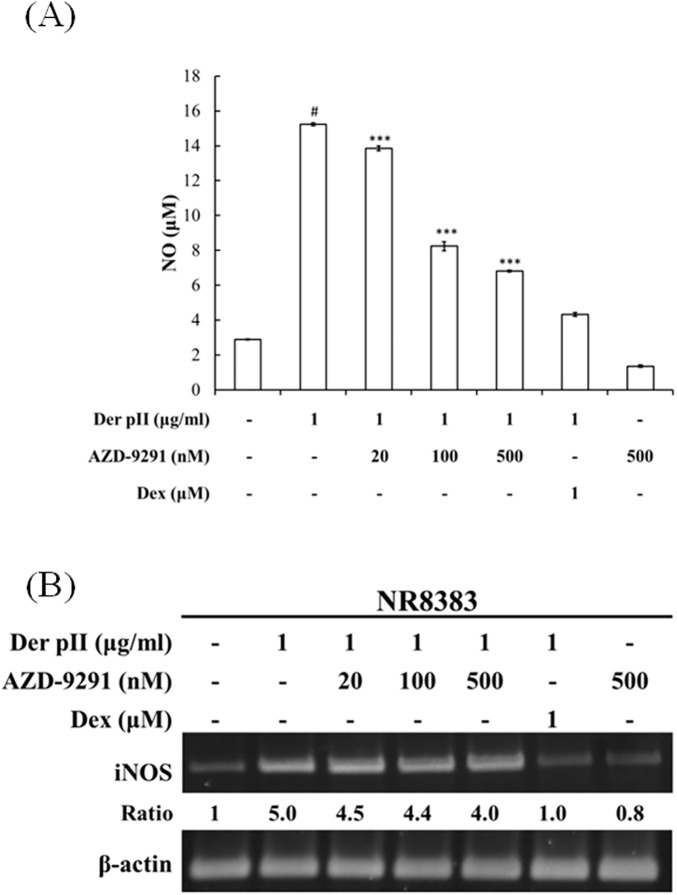
Effects of AZD-9291 on Der pII-induced nitrite oxide production in rat alveolar macrophages. The NR8383 cells (4 × 10^6^ cell/ 3.5 cm dish) were pre-treated with AZD-9291 (20, 100, 500 nM) or Dexamethasone (1 μΜ) for 2 h followed by stimulated Der pII (1 μg/mL) for 24 h. **(A)** Conditioned media was assayed level of NO by Griess reagent system. The data are expressed as mean ± SD. The symbols (#) indicated significant differences as compared with control, and (*) as compared with Der pII alone. *p < 0.05, **p < 0.01,***p < 0.001. **(B)** Gene expressions of INOS was analyzed by RT-PCR.

To investigate the effect of AZD-9291 on Der pII-induced stimulation of pulmonary alveolar macrophages, NR8383 cells—an alveolar macrophage cell line—were used as a model to assess NO production. As shown in Figure 6A, Der pII-induced NO production was significantly inhibited by AZD-9291. However, AZD-9291 only slightly reduced the gene expression of iNOS induced by Der pII.

## 4 Discussion

Inflammation involves both local manifestations, such as redness and swelling, and systemic responses, including fever. Neutrophils are recruited during the early stages of inflammation, followed by monocytes and lymphocytes in the later stages. Cytokines, including IL-17, stimulate stromal cells to produce IL-6 and IL-8. IL-6 primarily mediates fever, while IL-8 promotes the recruitment and accumulation of neutrophils in the blood and inflamed tissues (19–21).

Human peripheral blood mononuclear cells (HPBMCs), comprising lymphocytes and monocytes with a round nucleus, exhibited significantly reduced IL-6 and IL-8 secretion upon Der pII stimulation when treated with AZD-9291 or Tarceva (Figure 3). This indicates that AZD-9291 may be a promising multi-target anti-inflammatory agent, potentially useful in allergic airway diseases such as asthma, even in the absence of EGFR mutations.

In the present study, Der pII induced the secretion of IL-6 and IL-8 in THP-1 macrophages. AZD-9291 and Tarceva effectively suppresses the secretion of IL-6 and IL-8 induced by Der pII (Figure 5). Interestingly, Der pII stimulation in THP-1 macrophages leads to an upregulation of CD14 and CD36, and AZD-9291 treatment significantly inhibits the expressions of these genes. CD36, a class B scavenger receptor expressed in diverse cell types such as bone marrow cells, platelets, endothelial cells, and adipocytes, plays essential roles in fatty acid transport, angiogenesis inhibition, and bacterial phagocytosis (22, 23). CD14, a glycoprotein predominantly expressed on mature cells of the monocyte lineage, has been shown to mediate LPS-induced signaling pathways in macrophage phagocytosis (24). Upon bacterial infection, LPS binds to LPS-binding proteins and then form a complex with CD14, activating the TLR4-MyD88 signaling cascade (25). This pathway triggers the translocation of NF-*κ*B to the nucleus, leading to the transcription of pro-inflammatory genes such as TNF-α, IL-1β, IL-6, and iNOS (26).

In pulmonary alveolar macrophage models, Der pII stimulation increased NO production, a hallmark of inflammatory activation, but this was significantly reduced by AZD-9291. Interestingly, this reduction was not correlated with suppressed iNOS gene expression, suggesting that AZD9291 affects NO production at a post-transcriptional level or directly influences enzyme activity. Additionally, AZD-9291 reduced expression of macrophage activation markers CD14 and CD36 in THP-1 cells, indicating it may suppress macrophage polarization.

## 5 Conclusion

In this study, human peripheral blood mononuclear cells (HPBMCs), THP-1 monocytes, and THP-1–derived macrophages were used to investigate Der pII–induced inflammatory responses. We demonstrated that the EGFR inhibitors significantly suppressed Der pII–induced production of IL-6, IL-8, and nitric oxide in HPBMCs, monocytes, and macrophages. This is the first study to examine the regulatory effects of EGFR inhibitors on Der pII–induced inflammation in monocytes and macrophages.

## Data Availability

The original contributions presented in the study are included in the article/Supplementary Material, further inquiries can be directed to the corresponding author/s.
