# Supplementary material for: EGFR inhibitors suppress house dust mite allergen Der pII induced inflammation in monocytes and macrophages
Source: Front Allergy. 2026 Feb 6;7:1748679. doi: 10.3389/falgy.2026.1748679 (PMC12920423; doi:10.3389/falgy.2026.1748679)
Supplement: Supplementary file 1 [file Table1.docx]

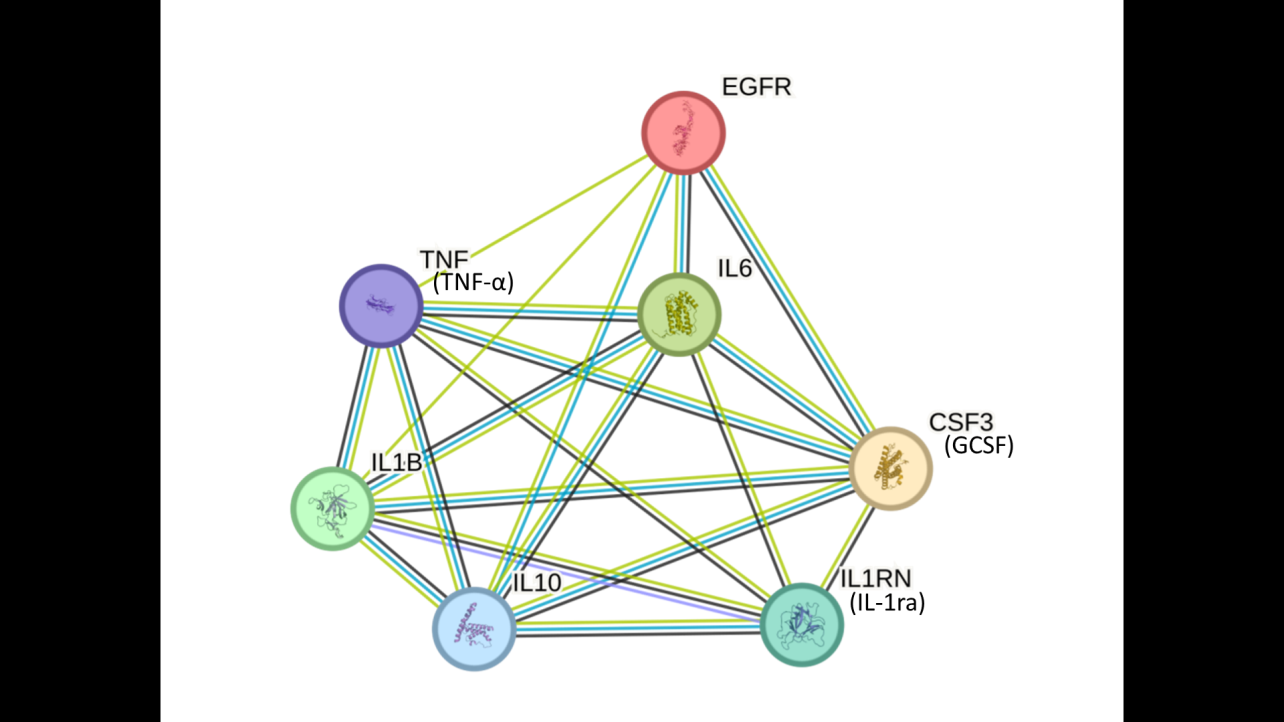


**Supplementary Figure 1.** The relationship between EGFR and the Der pII-induced cytokines was analyzed by STRING database.


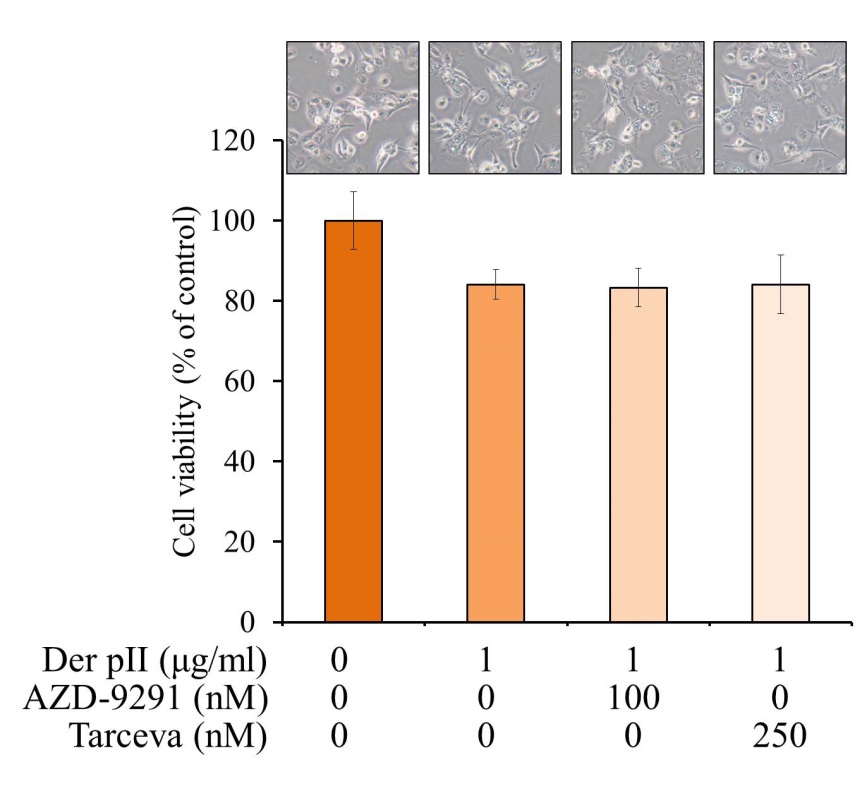


**Supplementary Figure 2. Effect of AZD-9291 or Tarceva on cell viability of THP-1 macrophage with Der pII treatment.**

THP-1 macrophage (1 x 10^6^ cell/3.5 cm dish) were pre-treated with 100 nM AZD-9291 or 250 nM Tarceva for 2 h followed by stimulated Der p II (1 μg/ml) for 24h. THP-1 macrophage were trypsinized, harvested, and counted using trypan blue staining.


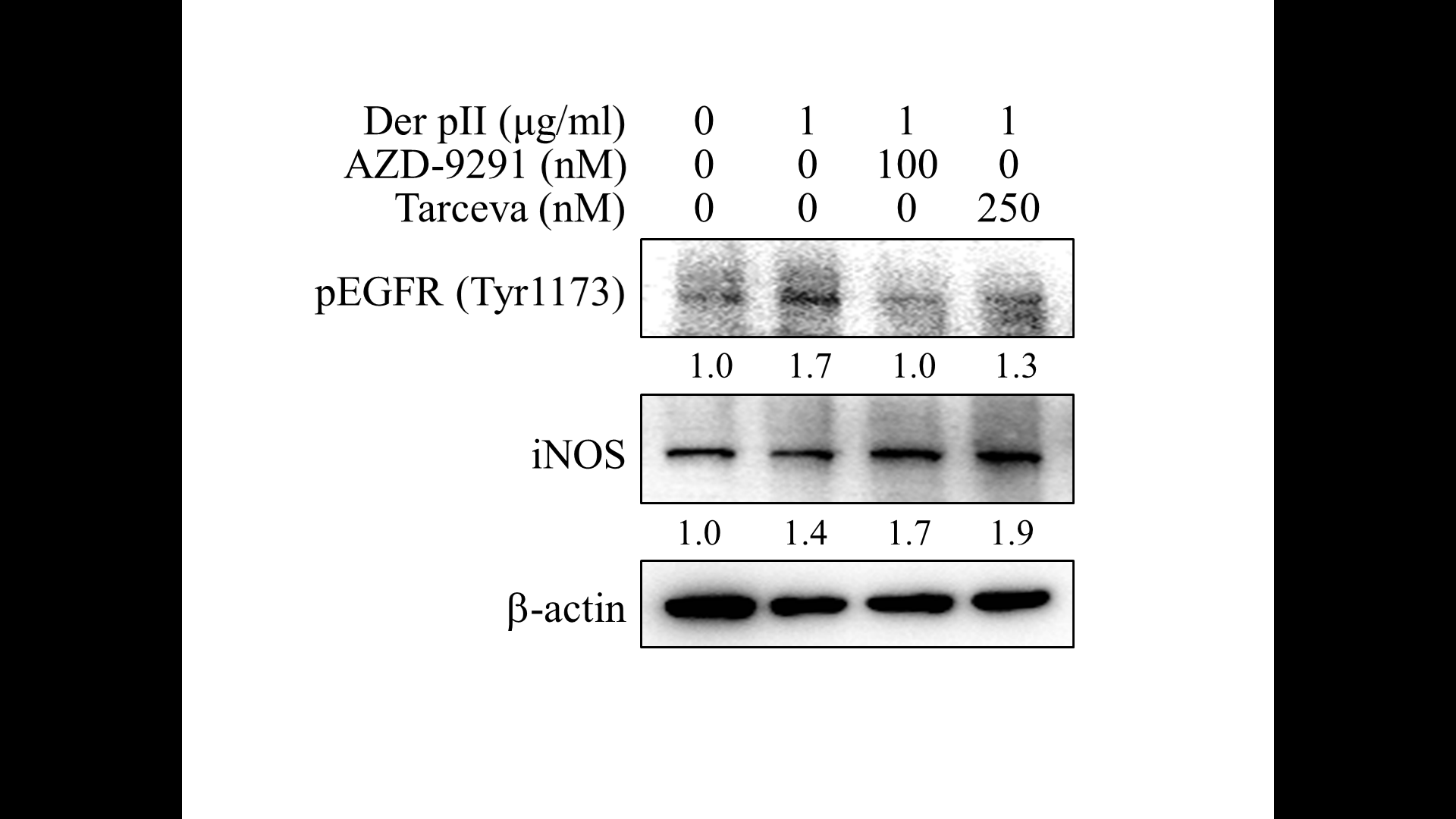


**Supplementary Figure 3. Effect of AZD-9291 or Tarceva on protein expressions of phospho-EGFR and iNOS in THP-1 macrophage with Der pII treatment.**

THP-1 macrophage (1 x 10^6^ cell/3.5 cm dish) were pre-treated with 100 nM AZD-9291 or 250 nM Tarceva for 2 h followed by stimulated Der p II (1 μg/ml) for 24h. Total cell lysates were used to investigate the indicated protein expression by Western blot assay. Quantify the band intensities were analyzed by ImageJ software. Data shown are the relative expression standardized by the β-actin protein level. The ratio of cells without treatment was set at 1.
